# Association between body composition standards and eating disorder medical claims among active-duty service women

**DOI:** 10.1186/s40337-024-00990-5

**Published:** 2024-02-19

**Authors:** Jessica Korona-Bailey, Amanda Banaag, Jasmine Walker, Stephanie Fortin, Megan Eyeler, Tracey Pérez Koehlmoos

**Affiliations:** 1 Center for Health Services Research, Uniformed Services University of the Health Sciences, 4301 Jones Bridge Road, Bethesda, MD 20814 USA; 2 The Henry M. Jackson Foundation for the Advancement of Military Medicine Inc., 6270A Rockledge Drive, Bethesda, MD 20817 USA

**Keywords:** Disordered eating, Body composition standards, Military Health System, Active duty service women, Women’s health

## Abstract

**Introduction:**

Eating disorders are a worldwide public health concern with the United States having a particularly high prevalence. Eating disorders are of particular concern to the Department of Defense and Military Health System (MHS) because body composition standards are in place for active-duty service members.

**Methods:**

We conducted a cross-sectional study of active-duty service women (ADSW) ages 18 and older in the U.S. Army, Air Force, Navy, and Marine Corps during fiscal years (FY) 2018–2019. Utilizing claims data from the MHS Data Repository (MDR), we identified ADSW with a Body Mass Index (BMI) measure during the study period and compared their BMI to Service-specific requirements and diagnosis of an eating disorder.

**Results:**

We identified a total of 161,209 ADSW from the MDR in FYs 2018–2019 with a recorded BMI, of whom 61,711 (38.3%) had a BMI exceeding the maximum BMI Service-specific standards during the study period and 0.5% had an eating disorder diagnosis. Increased risk of an eating disorder was found in ADSW with an Underweight BMI. Further, we found that there was no association of disordered eating diagnoses among ADSW who were near the maximum height/weight standard set by their Service.

**Conclusion:**

There appears to be no association between body composition standards of the Services and eating disorder diagnoses in ADSW. We were not able to investigate unhealthy habits around diet or exercise directly related to body composition standards.

**Supplementary Information:**

The online version contains supplementary material available at 10.1186/s40337-024-00990-5.

## Abstract (plain language summary)

Eating disorders are a worldwide public health concern with the United States having a particularly high prevalence. Active duty service women serving in the United States armed forces may be at an increased risk due to strict Service specific weight requirements. This study suggests that the height and weight standards do not increase the risk for eating disorder diagnoses in active duty service women. However, we were not able to investigate unhealthy habits around diet or exercise directly related to body composition standards near the time of measurement or assessment.

**Supplementary Information:**

The online version contains supplementary material available at 10.1186/s40337-024-00990-5.

## Introduction

Eating disorders are a worldwide public health concern with the United States (U.S.) having a particularly high prevalence [1, 2]. In the U.S., the prevalence of eating disorders ranges from 2.0 to 13.5% and studies show women to have a greater odds of lifetime diagnosis compared to men [1, 2]. Eating disorders are defined as disordered eating patterns or behaviors that can negatively impact physical and psychological health and manifest in a number of different diagnoses classified as anorexia nervosa, bulimia nervosa, and binge eating disorder. An additional disorder is “other specified feeding or eating disorders” which includes any type of abnormal eating behavior that impairs an individual’s social life [1–3].

Eating disorders can cause long term physical health consequences including malnutrition, overnutrition, gastrointestinal issues, endocrine and metabolic disorders, reproductive issues, cardiovascular problems, osteoporosis, and skin problems [4]. Comorbidities include mental health conditions such as depression, bipolar disorder, anxiety, suicidality, obsessive compulsive disorder, post-traumatic stress disorder (PTSD), substance use disorders, and sleep disturbances [4–6]. In fiscal year (FY) 2018–2019, total economic costs for eating disorders were estimated to be $64.7 billion. Costs of reduced wellbeing was valued at $326.5 billion leading to an urgency in identifying effective policy actions to reduce the impact of eating disorders [7].

Eating disorders are of particular concern to the Department of Defense (DoD) and Military Health System (MHS), which is charged with ensuring the health of the nation’s fighting force, of which, 17% are active-duty service women (ADSW) [8]. Some studies show that prevalence estimates of eating disorders in the U.S. military are similar to the general population, however other survey-based studies show higher prevalence [3, 9, 10]. A number of factors increase the risk of ADSW developing an eating disorder. For example, traumatic experiences are a strong risk factor [3, 11]. Additionally, limited evidence has shown that height and weight regulations, body composition standard, have encouraged unhealthy dieting behaviors by service members to meet standards [12, 13]. Each of the military service branches has instituted body mass index (BMI) standards to ensure a force that looks both professional and can physically perform their duties. From FY 2018–2019, BMI standards for the Army range from 25.0 to 26.0 and vary with age [14]. The Air Force has a BMI standard of 27.5. The Navy has a BMI standard that varies from 25.0 to 27.5 varying with height, and the Marine Corps has a single standard of 25.9 [15, 16]. Testing occurs twice a year and service members are penalized if they do not meet requirements.

Eating disorders can detrimentally impact a service member’s readiness as some individuals with the condition experience dizziness, fatigue, trouble concentrating, and electrolyte imbalances, all of which could endanger other service members and consume medical resources [3, 17]. As such, eating disorders were among the top behavioral health diagnoses with a high absolute risk of permanent profile for service members in the Army [18]. A permanent profile refers to a medical profile that outlines a soldiers’ physical limitations and restrictions which can potentially result in an end to military service. A more recent study found the incident rate of eating disorders among ADSW to be 13.8 per 10,000 person years between 2017 and 2021 [19]. The incident rate increased each year during the study period instilling the significance of the issue in this population [19]. While several studies have assessed prevalence or incidence of eating disorders in ADSW and female veterans, studies comparing eating disorder diagnoses to BMI standards are lacking. The aim of this study is to assess the prevalence of eating disorders in ADSW in FY 2018–2019 and associated risk factors, while also comparing BMI of ADSW to height and weight standards of the Services. We expect ADSW with a BMI close to the Service specific maximum standard to have increased prevalence of eating disorder diagnoses.

## Methods

### Data source and study design

We used the MHS Data Repository (MDR) to conduct a cross-sectional study of ADSW in the U.S. Army, Air Force, Navy, and Marine Corps during FYs 2018 to 2019. The MDR houses administrative and healthcare claims data for MHS beneficiaries including active-duty service members, retirees, and their dependents; however, claims data do not capture care delivered in combat zones or through the Veterans Health Administration [20]. The MDR does include claims captured by TRICARE in Military Treatment Facilities and private-sector facilities. Data from the MDR have been used in previous studies investigating health of ADSW [21, 22]. The study was considered exempt by the Institutional Review Board of the Uniformed Services University of the Health Sciences.

### Study population

Using the Defense Enrollment Eligibility Reporting System (DEERS) in the MDR, we identified all ADSW age 18 years and older from FYs 2018–2019. Women in the National Guard or Reserves, both active and inactive, were excluded due to their inconsistent access to care in the MHS. Additionally, we excluded pregnant women as well as 12-month postpartum women from our population. We limited our study sample to ADSW with a height and weight recorded on the medical record. BMI was calculated using the following metric system formula: (weight (lbs)/[height in inches (in)]^2^ × 703). The most recent and biologically plausible BMI measurement per patient was retained for analysis. Implausible BMI values were identified for exclusion if they were greater than ± 3 times the interquartile range and if recorded height values did not meet minimum accession standards for each Service. BMI of the study population was defined in relation to standards set by each Service branch during FY 2018–2019 (Additional file 1: Appendix Table S1). Service-specific BMI categories were defined first as below minimum BMI standards and exceeds maximum BMI standards. We created a borderline BMI defined as ± one BMI value above/below each Service’s maximum value. BMI classification was also defined using the following standard categorization: Underweight (< 18.5 kg/m^2^), Healthy weight (18.5–24.9 kg/m^2^), Overweight (25–29.9 kg/m^2^), and Obesity (≥ 30 kg/m^2^).

**Table 1:** ICD-10 codes and descriptions of eating disorder diagnoses

ICD-10 code	Condition description
F50.0	Anorexia nervosa: not maintaining normal body weight due to fear or disturbed perception of body image
F50.2	Bulimia nervosa: episodes of binge eating and behaviors to prevent weight gain included vomiting, laxative and diuretics use, fasting, and excessive exercise
F50.81	Binge eating disorder: episodes of eating large quantities of food often to the point of discomfort and loss of control
F50.89, F50.9	Other/unspecified eating disorders

Utilizing International Classification of Disease codes, 10th Revision (ICD-10), we identified ADSW with a diagnosis of an eating disorder. Disordered eating was defined as anorexia nervosa (F50.0), bulimia nervosa (F50.2), other categories such as binge eating disorder to and other or unspecified eating disorder (F50.81, F50.89 and F50.9) [23]. Full descriptions are in Table 1 below. A dichotomous variable was created in which the sample was categorized as either having and eating disorder diagnosis or not having an eating disorder diagnosis.

### Statistical analysis

The associations between eating disorder prevalence and several demographic characteristics including age, Service branch, marital status, race, and rank, a proxy for socioeconomic status were examined. Descriptive statistics were performed on patient demographics and Service-related characteristics (age group, race, military Service rank, branch of Service, BMI category, and Service BMI standard) for the total population and by eating disorder diagnosis. The prevalence of eating disorders in ADSW was calculated and expressed as a percentage. Group differences between ADSW with and without eating disorders were analyzed utilizing the chi-square test for independence. Unadjusted logistic regression analysis was performed on each categorical variable to assess their association with eating disorder diagnosis in ADSW. To control for confounding factors, a subsequent logistic regression was performed and adjusted by age, Service branch, marital status, race, and rank. Any observations with missing values were automatically removed from the logistic regression analyses. For all analyses, *p* values < 0.05 were considered statistically significant and were performed using SAS version 9.4.

## Results

We identified a total of 161,209 ADSW from the MDR in FYs 2018–2019, of whom 38.3% had a BMI exceeding their Service-specific maximum BMI standard during the study period, 61.7% had a BMI below the Service-specific maximum BMI standard, and 21.0% had a borderline BMI based on the most recent BMI recorded in their medical record. Table 2 details demographic distributions for the total ADSW study population and within group distributions by Service-specific BMI standard category during the study period. (Table 2) The majority of groups, including those with an eating disorder (52.5%), had a BMI below Service-specific standards based on their most recent BMI recorded in their medical record. However, ADSW aged 35–44 (51.3%), of Black race (51.1%), and of Senior Enlisted rank (50.5%) had a BMI exceeding the maximum Service-specific BMI standard based on the most recent BMI recorded. We identified 765 (0.5%) ADSW with an eating disorder diagnosis during the study period (Table 3). The diagnosis of binge eating disorder or unspecified/other eating disorder was the most frequently occurring diagnosis (100%) followed by anorexia nervosa (19.1%), and bulimia nervosa (2.7%). When assessing by demographic characteristics, the majority of ADSW were age 18–24 (50.2%), of White race (62.3%), unmarried (65.8%), and with a Healthy BMI (39.0%). The borderline Service-specific BMI standard category accounted for 14.6% of eating disorder diagnoses. For Service-specific factors, the majority of ADSW with an eating disorder were in the Army (34.0%) and were a Junior Enlisted rank (48.8%).

**Table 2:** Demographics of ADSW Study Population by Status of Meeting Service-specific Maximum BMI Standards, FY 2018–2019

	Total study population	Below maximum BMI standards	Exceeds maximum BMI standards	Borderline BMI
		N (row %)	N (row %)	N (row percent of total pop %)
Total	161,209	99,498 (61.7)	61,711 (38.3)	33,790 (21.0)
Age group (years)				
18–24	76,584	52,686 (68.8)	23,898 (31.2)	16,287 (21.3)
25–34	53,902	31,727 (58.9)	22,175 (41.1)	11,265 (20.9)
35–44	24,568	11,968 (48.7)	12,600 (51.3)	4940 (20.1)
45–54	5678	2848 (50.2)	2830 (49.8)	1200 (21.1)
> = 55	477	269 (56.4)	208 (43.6)	98 (20.6)
Race				
White	93,224	61,635 (66.1)	31,589 (33.9)	20,012 (21.5)
Black	42,385	20,714 (48.9)	21,671 (51.1)	8980 (21.2)
Asian/Pacific Islander	11,662	7784 (66.8)	3878 (33.3)	2273 (19.5)
American Indian/Alaskan native	1892	1100 (58.1)	792 (41.9)	444 (23.5)
Other	6792	4172 (61.4)	2620 (38.6)	1271 (18.7)
Missing	5254	4093 (77.9)	1161 (22.1)	810 (15.4)
Marital status				
Married	60,194	34,492 (57.3)	25,702 (42.7)	12,587 (20.9)
Unmarried	101,015	65,006 (64.4)	36,009 (35.7)	21,203 (21.0)
Service				
Army	57,791	30,550 (52.9)	27,241 (47.1)	14,197 (24.6)
Air force	53,848	37,165 (69.0)	16,683 (31.0)	9134 (17.0)
Navy	36,255	21,182 (58.4)	15,073 (41.6)	6910 (19.1)
Marine corps	13,315	10,601 (79.6)	2714 (20.4)	3549 (26.7)
Rank				
Junior enlisted	78,322	51,112 (65.3)	27,210 (34.7)	17,010 (21.7)
Senior enlisted	47,733	23,637 (49.5)	24,096 (50.5)	9888 (20.7)
Junior officer	25,527	17,960 (70.4)	7567 (29.6)	5117 (20.1)
Senior officer	5639	3386 (60.1)	2253 (40.0)	1139 (20.2)
Other	3988	3403 (85.3)	585 (14.7)	636 (16.0)
Eating disorder diagnosis				
No	160,442	99,095 (61.8)	61,347 (38.2)	33,678 (21.0)
Yes	767	403 (52.5)	364 (47.5)	112 (14.6)

Borderline population includes service women from the below maximum BMI standard and exceeds maximum BMI standard categories

*ADSW* active duty service women

**Table 3:** Demographics of ADSW Study population by eating disorder status and chi-square *p*-values from difference in frequency tests, FY 2018–2019

	Total study population	No eating disorder diagnosis	Eating disorder diagnosis	Chi-square tests
	N (col %)	N (col %)	N (col %)	*p*-value
Total	161,209	159,967	765	
Age group (years)				0.26
18–24	76,584 (47.51)	76,199 (47.49)	385 (50.20)	
25–34	53,902 (33.44)	53,661 (33.45)	241 (31.42)	
35–44	24,568 (15.24)	24,447 (15.24)	121 (15.78)	
45–54	5678 (3.52)	5660 (3.53)	18 (2.35)	
> = 55	477 (0.30)	*	*	
Race				0.013
White	93,224 (57.83)	92,746 (57.81)	478 (62.32)	
Black	42,385 (26.29)	42,203 (26.30)	182 (23.73)	
Asian/Pacific Islander	11,662 (7.23)	11,626 (7.25)	36 (4.69)	
American Indian/Alaskan native	1892 (1.17)	*	*	
Other	6792 (4.21)	6761 (4.21)	31 (4.04)	
Missing	5254 (3.26)	5220 (3.25)	34 (4.43)	
Marital status				0.07
Married	60,194 (37.34)	59,932 (37.35)	262 (34.16)	
Unmarried	101,015 (62.66)	100,510 (62.65)	505 (65.84)	
Service				< 0.001
Army	57,791 (35.85)	57,530 (35.86)	261 (34.03)	
Air force	53,848 (33.40)	53,628 (33.43)	220 (28.68)	
Navy	36,255 (22.49)	36,046 (22.47)	209 (27.25)	
Marine corps	13,315 (8.26)	13,238 (8.25)	77 (10.04)	
Rank				0.14
Junior enlisted	78,322 (48.58)	77,948 (48.58)	374 (48.76)	
Senior enlisted	47,733 (29.61)	47,492 (29.60)	241 (31.42)	
Junior officer	25,527 (15.83)	25,423 (15.85)	104 (13.56)	
Senior officer	5639 (3.50)	5617 (3.50)	22 (2.87)	
Other	3988 (2.47)	3962 (2.47)	26 (3.39)	
BMI category				< 0.001
Underweight	1318 (0.82)	1294 (0.81)	24 (3.13)	
Healthy	73,141 (45.37)	72,842 (45.40)	299 (38.98)	
Overweight	64,176 (39.81)	63,945 (39.86)	231 (30.12)	
Obesity	22,574 (14.00)	22,361 (13.94)	213 (27.77)	
Body composition status				< 0.01
Below maximum BMI standards	99,498 (61.72)	99,095 (61.76)	403 (52.54)	
Exceeds maximum BMI standards	61,711 (38.28)	61,347 (38.24)	364 (47.46)	
Borderline BMI	33,790 (20.96)	33,678 (20.99)	112 (14.60)	< 0.01

*ADSW* active duty service women, *BMI* body mass index

^*^Censored due to one or more stratified cell counts < 11

Table 4 shows unadjusted and adjusted logistic regression results characteristics of ADSW associated with an eating disorder diagnosis during the study period (Table 4). After adjustment for all variables included in the model, logistic regression results indicate there is no association between having a BMI within the borderline limits of Service-specific standards and having an eating disorder diagnosis (*p* > 0.05). While having an overweight BMI was not significantly associated with an eating disorder (*p* > 0.05), we observed higher odds of an eating disorder in ADSW in an underweight BMI category (aOR 4.48, CI 2.89–6.95) and the obesity BMI category (aOR 2.21, CI 1.57–3.11) compared to those with a Healthy BMI. With regards to demographic and Service characteristics, logistic regression results indicate lower odds of an eating disorder in ADSW of Asian/Pacific Islander race (aOR 0.61, CI 0.43–0.85), Black race (aOR 0.74, CI 0.62–0.89), and in the Air Force (aOR 0.82, CI 0.68–1.00) compared to those of White race and in the Army. No significant associations were observed for age, marital status or rank (*p*’s > 0.05).

**Table 4:** Unadjusted and adjusted logistic regression results for odds of an eating disorder, FY 2018–2019

Effect	Unadjusted				Adjusted			
	OR	95% CI		*p*-value	OR	95% CI		*p*-value
Age group (years)								
18–24 (ref)	1.00	1.00	1.00		1.00	1.00	1.00	
25–34	0.89	0.76	1.05	0.1525	0.88	0.71	1.09	0.2283
35–44	0.98	0.80	1.20	0.8436	0.93	0.69	1.24	0.6092
45–54	0.63	0.39	1.01	0.0553	0.62	0.36	1.07	0.0837
55 and older	0.83	0.21	3.35	0.7975	0.83	0.20	3.55	0.805
Race								
White (ref)	1.00	1.00	1.00		1.00	1.00	1.00	
Black	0.84	0.71	0.99	0.0412	0.74	0.62	0.89	0.001
Asian/Pacific Islander	0.60	0.43	0.84	0.0032	0.61	0.43	0.85	0.004
American Indian/Alaskan native	0.62	0.28	1.38	0.241	0.56	0.25	1.26	0.1632
Other	0.89	0.62	1.28	0.5291	0.83	0.57	1.20	0.31
Marital status								
Married (ref)	1.00	1.00	1.00		1.00	1.00	1.00	
Unmarried	1.15	0.99	1.34	0.0682	0.86	0.74	1.02	0.0763
Service								
Army (ref)	1.00	1.00	1.00		1.00	1.00	1.00	
Air force	0.90	0.76	1.08	0.2727	0.82	0.68	1.00	0.0468
Navy	1.28	1.07	1.53	0.0083	1.11	0.91	1.35	0.2945
Marine corps	1.28	0.99	1.66	0.0558	1.30	1.00	1.70	0.0526
Rank								
Junior enlisted	1.23	0.80	1.89	0.3558	0.93	0.55	1.57	0.7916
Senior enlisted	1.30	0.84	2.01	0.2458	1.06	0.65	1.72	0.8289
Junior officer	1.04	0.66	1.66	0.8533	0.88	0.53	1.47	0.6237
Senior officer (ref)	1.00	1.00	1.00		1.00	1.00	1.00	
Other	1.68	0.95	2.96	0.0756	1.33	0.57	3.08	0.5105
BMI Category								
Underweight	4.52	2.97	6.87	< .0001	4.48	2.89	6.95	< .0001
Healthy (ref)	1.00	1.00	1.00		1.00	1.00	1.00	
Overweight	0.90	0.78	1.05	0.1748	0.86	0.67	1.11	0.2396
Obesity	2.32	1.95	2.77	< .0001	2.21	1.57	3.11	< .0001
BMI body composition status								
Exceeds max BMI standards	1.46	1.27	1.68	< .0001	1.28	1.00	1.64	0.0462
Borderline BMI*	0.64	0.53	0.787	< .0001	0.82	0.65	1.03	0.0911
Below BMI standards	1.00	1.00	1.00		1.00	1.00	1.00	

*Borderline BMI status was included in a separate multivariate model from below/exceeds max BMI standards due to overlap between the two variables. In the adjusted model, adjusted confounders include: age group, race, Service branch, rank, and BMI category

*ADSW* active duty service women, *BMI* body mass index

## Discussion

This cross-sectional study identified 161,209 ADSW with a recorded BMI from FY 2018 to 2019 serving in the US Army, Air Force, Navy and Marine Corps. Most ADSW, 61% were below the maximum Service-specific BMI standards. Prevalence of eating disorder diagnosis was low at 0.5%. The highest prevalence of eating disorder diagnosis for each category was in ADSW of White race, unmarried, serving in the Army, and of Junior enlisted rank. Odds of an eating disorder diagnosis were increased for ADSW with Underweight BMI or a BMI assigned as obesity and no association was found between a borderline Service-specific BMI and eating disorder diagnosis.

Prevalence of eating disorders was low in this study at 0.5% compared to some prevalence ranges of 2–13.5% in civilian populations [1, 2]. However, when limiting the comparison to studies using claims data for identification, our study is in line with civilian estimates of 0.3% and military estimates of 0.6% [1–3]. The differences in estimates are important to note when studying prevalence of eating disorders. Using medical claims for identification has the added benefit of documented diagnosis by a medical professional. An alternate approach to identification is using self-report assessments following up with interview assessments to confirm diagnoses which is more likely to identify perspectives from patients whether or not they have sought care for a condition. Studies using this two-stage approach report similar estimates of 0.2–1.7% in civilian populations [1, 3].

There is a pervasive belief that military weight standards may contribute to increased risk factors for disordered eating behaviors around the time of height and weight measures and tape tests. Several studies have investigated this hypothesis and found increased prevalence near testing periods [13, 24]. One study of active-duty personnel assigned to a Navy hospital had prevalence ranging from 5 to 18%, with diet pills, diuretics, and laxatives being the most common behaviors [24]. However, this study was a self-report survey and the response rate was low which could skew results [24]. In a study published by Antczak and colleague in 2008, the Marines had the majority (66%) of anorexia nervosa diagnosis, and females, specifically White females, had a higher incidence of eating disorders [25]. A study looking at incidence rates over a 5-year period found higher incidence of eating disorder diagnoses in women under age 30, White race, serving in the Marine Corps, and of Junior Enlisted rank [19]. Our study showed a similar trend in prevalence of eating disorder diagnoses among White females (62%), ADSW under age 30, and ADSW of Junior Enlisted rank. However, service in the Marine Corps did not yield a significant finding when comparing with other branches of Service.

While our study did not assess the diagnosis around a testing period, we compared BMI as recorded in the medical record to the Service-specific BMI standards during the study period and found no association between a borderline BMI category and an eating disorder diagnosis. Overall, the BMI category with the largest risk for an eating disorder was ADSW who were Underweight. A study by Carlton et al. discussed the idea that some new recruits may present to training with pre-existing subclinical disordered eating attitudes and behaviors despite being examined at the Military Entrance Processing Station [24]. We cannot be sure whether the ADSW in our study had an eating disorder prior to joining the armed forces or if they developed one during their service time. Eating disorder diagnosis prior to accession is highly limited and based on self-report of past or current diagnoses and not necessarily current symptoms.

The DoD as a whole has undergone a complete overhaul of the physical fitness and body composition program brought on by DoD Instruction 1308.03 in 2022 in an effort to improve the health and well-being of service members [26]. The new BMI standard is a maximum of 27.5 which was the maximum for the Air Force in this study. In recent years, the Air Force and Army have adjusted body compositions measurements for their tape test policies [27, 28]. Additionally, the Army and Marines Corps have implemented directives where Soldiers and Marines who score at a certain level on physical fitness tests are exempt from body composition assessment [28, 29]. Body composition requirements are important to maintain a ready force. While some service members may engage in risky behaviors to meet body composition requirements, our study demonstrates that ADSW with a borderline BMI measurement are not at increased risk for eating disorder diagnosis. Increasing the body composition maximum limits as the Services have done can allow service members to gain muscle mass to meet physical fitness requirements. Follow on research should be conducted to determine which body composition standards are appropriate for ADSW to achieve the physical fitness requirements of their Service branch, which was a recommendation in a Defense Health Board report discussing ADSW’s health care services [30].

## Limitations

This study had several limitations. The use of claims data have the potential for coding errors and inadequate specificity for a condition. There are limitations in the granularity of race data that we were able to report. Additionally, we recognize that race is not a sufficient proxy for discrimination and prejudice that occurs in healthcare. Further, we did not capture undiagnosed disordered eating behavior. An eating disorder diagnosis may be stigmatized with negative career impacts and ADSW may not seek professional care for their condition. Additionally, this study does not capture data for any healthcare received outside of the TRICARE benefit.

## Conclusion

Our study found no association between body composition standards of the Services and disorder eating diagnosis among ADSW. The highest risk for eating disorders occurred in ADSW who were Underweight. Future research should examine how nutritional education programs can be designed in initial entry training to address unhealthy eating attitudes and behavior to identify those who may be entering into the Service with an underlying condition. Additionally, future research should aspire to better understand dieting and unhealthy eating behaviors around the time of body composition assessments and determine ways to mitigate such behavior.

### Supplementary Information

**Additional file 1**. **Appendix Table S1**: Maximum Body Mass Index Cutoffs by Service Branch and Year.

## Data Availability

The data that support the findings of this study are available from the Department of Defense via the Defense Health Agency, but restrictions apply to the availability of these data, which were used under license for the current study, and so are not publicly available. Data are however available from Dr. Tracey Koehlmoos, upon reasonable request and with permission of the Defense Health Agency.

## Abbreviations

aOR: Adjusted odds ratio
BMI: Body mass index
DEERS: Defense enrollment eligibility reporting system
DoD: Department of defense
FY: Fiscal year
ICD-10: Utilizing International Classification of Disease codes, 10th Revision
MDR: Military Health System Data Repository
MEPS: Military Entrance Processing Station
MHS: Military Health System
OR: Odds ratio
PTSD: Post traumatic stress disorder
US: United States
