# Supplementary material for: Association between body composition standards and eating disorder medical claims among active-duty service women
Source: J Eat Disord. 2024 Feb 19;12:29. doi: 10.1186/s40337-024-00990-5 (PMC10875869; doi:10.1186/s40337-024-00990-5)
Supplement: Supplementary file 1 — Additional file 1. Appendix Table S1: Maximum Body Mass Index Cutoffs by Service Branch and Year. [file 40337_2024_990_MOESM1_ESM.docx]

Appendix Table 1. Maximum Body Mass Index Cutoffs by Service Branch and Year

| Military Service Branch | Different Categories | Body Mass Index Cutoff | Year |
| --- | --- | --- | --- |
| Army | Age <= 20 | 25.0 | 2013 |
|  | Age 21-27 | 25.3 |  |
|  | Age 28-39 | 25.6 |  |
|  | Age => 40 | 26.0 |  |
| Air Force | - | 27.5 | 2019 |
| Navy | Height <= 61 inches | 27.5 | 2016 |
|  | Height 62-65 inches | 27.0 |  |
|  | Height 66-69 inches | 26.0 |  |
|  | Height 70-71 inches | 25.5 |  |
|  | Height =>72 inches | 25.0 |  |
| Marine Corp | - | 25.9 | 2016 |
